# Analysis of 5-Methylcytosine Regulators and DNA Methylation-Driven Genes in Colon Cancer

**DOI:** 10.3389/fcell.2021.657092

**Published:** 2022-01-31

**Authors:** Cheng Du, XinLi Liu, Mingwei Li, Yi Zhao, Jie Li, Zhikang Wen, Min Liu, Meina Yang, Boshi Fu, Minjie Wei

**Affiliations:** ^1^ Department of Pharmacology, School of Pharmacy, China Medical University, Shenyang, China; ^2^ Liaoning Key Laboratory of Molecular Targeted Anti-Tumor Drug Development and Evaluation, Liaoning Cancer Immune Peptide Drug Engineering Technology Research Center, Shenyang, China; ^3^ Key Laboratory of Precision Diagnosis and Treatment of Gastrointestinal Tumors, Ministry of Education, China Medical University, Shenyang, China; ^4^ Department of Digestive Oncology, Cancer Hospital of China Medical University, Shenyang, China

**Keywords:** 5mC regulators, methylation-driven gene, colon cancer, diagnosis, biomarker

## Abstract

**Background:** Epigenetic-driven events are important molecular mechanisms of carcinogenesis. The 5-methylcytosine (5mC) regulators play important roles in the methylation-driven gene expression. However, the effect of the 5mC regulators on the oncogenic pathways in colon cancer (CC) remains unclear. Also, the clinical value of such epigenetic-driven events needs further research.

**Methods:** The transcriptome and matching epigenetic data were obtained from The Cancer Genome Atlas dataset. The gene set variation analysis identified the oncogenic pathways adjusted by 5mC regulators. The “edgeR” and “methylmix” package identified the differential expression genes of DNA methylation-driven genes. The correlation between 5mC regulators or transcription factors and shortlisted genes was investigated by calculating the Spearman's rank correlation coefficient. Among them, the genes related to diagnosis were screened out based on differential gene expression in extracellular vesicles (EVs) by the “limma” package and histology by immunohistochemistry. Then, a risk signature was constructed by fitting the generalized linear model and validated by the receiver operating characteristic curve.

**Results:** MYC targets pathway and phosphatidylinositol-3-kinase–AKT–mammalian target of rapamycin signaling pathway were identified as the hallmark-related pathways associated with 5mC regulators. Also, the P53 pathway was subject to the influence of regulators' expression. A five methylation-driven gene signature (FIRRE, MYBL2, TGFBI, AXIN2, and SLC35D3) was developed as the biomarker for CC diagnosis. Meanwhile, those genes positively related to 5mC regulators and interacted with their relevant or transcription factors.

**Conclusion:** In general, 5mC regulators are positively related to each other and DNA methylation-driven genes, with the relationship of multiple active and inhibitory pathways related to cancer. Meanwhile, the signature (FIRRE, MYBL2, TGFBI, AXIN2, and SLC35D3) can prefigure prospective diagnosis in CC.

## Introduction

Colon cancer (CC) is one of the most common malignancies with heterogeneous incidence and mortality around the world (Siegel et al., 2021). During the last decade, the link between molecular events and clinical characteristics, including prognosis and therapeutic responses, has been at critical attention (Fearon, 2011). Such molecular characteristics are the major contributor to biomarkers for clinical incoming. Currently, the growing academic interest in multi-omics has been witnessed. To date, there remains a paucity of systematic analyses focusing specifically on methylation-driven events.

DNA methylation, one common epigenetic modification in mammalians (Suzuki and Bird, 2008), is regulated by DNA methylation regulators. Methylation-driven events were molecular events regulated by DNA methylation. DNA methylation at position C-5 of the cytosine loop [5-methylcytosine (5mC)] is the most common epigenetic event with the dynamic modification character (Bestor et al., 2015). The enzymes associated with hypermethylation or demethylation have been identified as the 5mC regulators. They are always grouped into three categories: “writers,” “erasers,” and “readers.” “Writers” (DNA methyltransferases) can modify methylation on peculiar nucleotide bases in consort with “erasers” (DNA demethylase), eliminating these marks, and “readers” can recognize methylated DNA and provide the competence in recruiting other factors (Moore et al., 2013).

Poh et al. (2016) and Zhang and Xu (2017) illustrated that DNMT3A and DNMT3B are essential for *de novo* methylation of the genome to keep a cell's specific methylation profile and also for methylation of newly integrated retroviral sequences. “Erasers” [ten–eleven translocation (TET) family proteins] can remove DNA methylation and mediate the conversion of oxidized 5mC to 5-hydroxymethylcytosine in an α-ketoglutarate- and Fe(II)-dependent manner (Tahiliani et al., 2009). Screening of tumor suppressor genes in metastatic colorectal cancer (CRC) has revealed that methyl-binding protein 1 (MBD1) gene, compared with other genes that were screened for methylation levels, had the most downregulated messenger RNA (mRNA) expression and upregulated methylation levels in advanced CRC (Parry and Clarke, 2011). Some research has demonstrated that DNA methylation plays an important role in various biological processes (Luo et al., 2018), such as attaching transcriptional regulation (Poh et al., 2016) and functioning in diverse genomic partitions and developmental stages (Zhou et al., 2018). Li et al. (2020) proposed that upregulated writers (DNMT1, DNMT3A, and DNMT3B) facilitated hypermethylation and downregulation of BAD and INPPL1 in colitis-associated cancer. Xu et al. (2019) recommended TET2 (eraser) activity as a biomarker to predict the efficacy of anti-PD-1/PD-L1 treatment and patient response and the stimulation of TET2 activity as adjunct immunotherapy for CC. The MBD2 (reader) antigen only reacted with the serum of CC patients but not with the serum of normal blood donors (Martin et al., 2008). In summary, these results show that 5mC regulators play an important role in CC.

Specific epigenetic events also have been demonstrated as effective biomarkers in CC (Hao et al., 2017). Based on the DNA methylation level, Ren et al., 92016) unearthed three major subtypes (MCL1, MCL2, and MCL3), which is helpful to understand the subtypes of CC. Bai et al. (2020) put forward that circulating DNA and its methylation level can be regarded as new indicators for colitis cancer transformation (Jones and Baylin, 2007). Ginder and Williams (2018) reviewed the possibility of the MBD family as potential therapeutic targets. Recently, Chen et al. (2020) identified cross talk between m6A and 5mC regulators across 33 cancer types, which connect two different methods of epigenetic regulation.

Extracellular vesicles (EVs) include exosomes, microvesicles, which is formed by direct plasma membrane budding and range from 100 to 1,000 nm in diameter (Thery et al., 2018), and exosomes are nano-sized vesicles (30–150 nm) originating from the endocytic pathway (Kowal et al., 2016). All EVs enclose or expose on their surface a multitude of biomolecules, including RNA, lipids, proteins, and possibly DNA, that can be trafficked between cells as a means of intercellular communication at both paracrine and systemic levels. The versatility of EVs in cancer made them contribute to many of the hallmarks of cancer (Meehan and Vella, 2016), including cell proliferation and migration (Kim et al., 2018), angiogenesis (Webber et al., 2015), evasion of cell death (Wieckowski et al., 2009), and invasion and metastasis (Fong et al., 2015). EVs have emerged as novel players in the prevention and treatment of various human diseases. Li et al. (2021a) showed a circulating EV long RNA-based signature for prognostic assessment of pancreatic ductal adenocarcinoma. Recently, Wu et al. (2021) found a diagnosis protocol to realize ultrasensitive detection of urinary EVs and accurate classification of early urinary diseases *via* radiometric three-dimensional DNA machine combined with a machine learning algorithm. In recent years, people have taken advantage of the dynamic behaviors in cells to develop advanced drug delivery systems; EVs have great potential as drug delivery vectors (Chen et al., 2021). An adequate therapeutic system was developed for the treatment of autoimmune encephalomyelitis using EVs from modified neural stem cells with high expressed ligand platelet-derived growth factor subunit A and achieving locally targeted delivery (Xiao et al., 2022).

In this study, we investigated the interrelation of canonical 23 5mC regulators in CC. The somatic genomic alterations and copy number alterations (CNAs) of such 5mC regulators were profiled. Then, the effect of the expression of 5mC regulators on oncogenic pathways was analyzed by gene set variation analysis and visualized by the Cytoscape software. This study inquired about genes driven by DNA methylation and performed enrichment analysis to investigate the impact of DNA methylation on genes. The approach to preferably identifying the clinical relevance of these genes adopted for this study was analyzing the expression in EVs and histology. This study utilized prospective genes to assess the relationship between genes and transcription factors (TFs) and 5mC regulators.

## Materials and Methods

### Data Collection

Multiple data of CC, including transcriptome expression, DNA methylation data analyzed by Illumina Human Methylation 450k, and mutation annotation format were acquired from The Cancer Genome Atlas (TCGA) database (https://portal.gdc.cancer.gov). We collected 430 cancer samples and 40 normal tissue samples in the transcriptome dataset and 316 cancer samples and 38 normal tissue samples in the epigenetic dataset. The processed result analyzed by GISTIC2.0 of copy number was downloaded from the Xena platform (https://xenabrowser.net). GSE39582 was collected from the Gene Expression Omnibus database.

### Collection and Analysis of 5-Methylcytosine Regulators

By reading pieces of literature published, 23 DNA methylation regulators were found to participate in the next research, including four writers, three erasers, together with 16 readers. Interconnection among DNA methylation regulators was resolved based on the analysis of the STRING database (http://www.stringdb.org/). The Shapiro–Wilk normality test was conducted to judge whether the expression of those genes conforms to normal distribution or not. The correlation analysis among DNA methylation regulators was performed with the R software.

### Cancer-Related Pathways Analysis of 5-Methylcytosine Regulators

To find cancer hallmark-related pathway activity along with expression efficiency of regulators in CC, gene set variation analysis (Hanzelmann et al., 2013), a nonparametric, unsupervised method for assessing gene set enrichment variation through gene expression profile, was performed according to the package instruction. These pathways with |log_2_FC| > 0.2 and *p*-value < 0.05 were regarded as significant enrichment pathways.

### Relationship Among Genes

With a view to the evaluation of the correlation among genes, the Shapiro–Wilk normality test was conducted to judge whether the expression of genes conformed to normal distribution. Further to the result, the “corrplot” package (https://github.com/taiyun/corrplot) was performed to calculate the Spearman correlation coefficient and *p*-value by R software. Cytoscape software was performed to visualize the interactions between 5mC regulators and pathways.

### Access to DNA Methylation-Driven Genes

The “edgeR” package (Robinson et al., 2010) in R software was used to normalize RNA sequencing data and then screened out the differential genes using the quasi-likelihood *F* test. We, as a result, selected the differential genes with |log_2_FC| > 2.0 and *p-*value < 0.05 for further research. The “methylmix” package (Cedoz et al., 2018) was used to build a β-mixed model whose CorThreshold was set to 0.4. Samples from TCGA involving 462 cancer tissues and 41 normal tissues were applied to preparation for methylation-driven genes with |DM-values| > 0 (differential methylation values). Finally, genes in line with differential methylation expression filtered by the “limma” package (Ritchie et al., 2015) with a *p-*value < 0.05 and |log_2_FC| > 0.1 were identified for the next investigation.

### Visualization of DNA Methylation-Driven Genes

“OmicCircos” package (Hu et al., 2014) was used to map the multi-omics profile of DNA methylation-driven genes from CC in TCGA database screened by the β mixed model, including corresponding chromosomal location, bar charts of DNA methylation degree, and dot plots of transcriptome expression degree.

### Gene Enrichment Analysis

The “ClusterProfiler” package (Yu et al., 2012) was used to perform gene set enrichment analysis of gene ontology (GSEA.GO) involving BP (Biological Process), CC (Cell Component) and MF (Molecular Function), and Kyoto Encyclopedia of Genes and Genomes (KEGG) pathway analysis on DNA methylation-driven genes with the filter criteria of the cutoff *p-*value that was set to 0.05.

### Clinical Relevance of 5-Methylcytosine Regulators

To deliver significant 5mC regulators of survival assessment in CC, The Human Protein Atlas (HPA) project database (https://www.proteinatlas.org/) was retrieved to find the best cutoff value of gene expression and draw Kaplan–Meier (KM) curves, based on 5-year survival condition. The drug–protein interaction information concerning regulators was collected with the “maftools” package (Mayakonda et al., 2018).

### Clinical Significance Assessment of DNA Methylation-Driven Genes

To sort out significant genes that can be regarded as prospective diagnostic biomarkers of CC, we did the following tasks. The first part was to find the optimal genes from the BBCancer database (http://bbcancer.renlab.org/), which should be apposite to the differential expression prerequisite in EVs or circulating tumor cells. Secondly, the “ROCR” package (Sing et al., 2005) using R statistical software was operated to calculate the area under the receiver operating characteristic curve (AUC) for them to measure and compare the model performance. Next, these genes conforming to the statistical result of *p-*value < 0.05 and AUC > 0.7, and the property of universal probes were worthy of further investigation on fitting generalized linear models using iteratively reweighted least-squares. Finally, the model based on iteratively reweighted least-squares could be concluded to construct a signature formula to help us predict CC. Also, the Z test was used to compare the AUC of biomarkers further. To find the optimal cutoff value, the point to be closest to the true-positive rate of (1) and the false-positive rate of (0) is acceptable, which is in the sense of equal trade-off between “sensitivity” and “specificity.”

### Immunohistochemistry-Based Expression Data of The Cancer Genome Atlas Samples

HPA is a systematic study to allow for a systematic exploration of the human proteome using antibody-based proteomics for multiple tissues and cell lines, which is accomplished by combining high-throughput generation of affinity-purified antibodies with protein profiling in a multitude of tissues and cells assembled in tissue microarrays. To compare candidates between pathology and normal immunohistochemistry-based expression data of TCGA samples, HPA was explored to collect and observe the immunohistochemistry results of colon adenocarcinoma tissue and colon normal tissue.

### Discovery of Transcription Factors Corresponding to Genes

TFs of genes with clinical benefits were explored and visualized with a web tool NetworkAnalyst (http://www.networkanalyst.ca), which can analyze comprehensive gene expression profiling, referring to ENCODE (Encyclopedia of DNA Elements) whose prerequisite was that betweenness was set to 2.

### Inherent Character of Genes in Colon Cancer

An inquiry into somatic genomic alterations and somatic mutation interactions was performed with the “Maftools” package to detect the results of somatic variants and significant pairs of genes, including 5mC regulators and the prospective diagnostic genes.

## Results

### Oncogenic Pathways Regulated by 5-Methylcytosine Regulators

**FIGURE 1:**
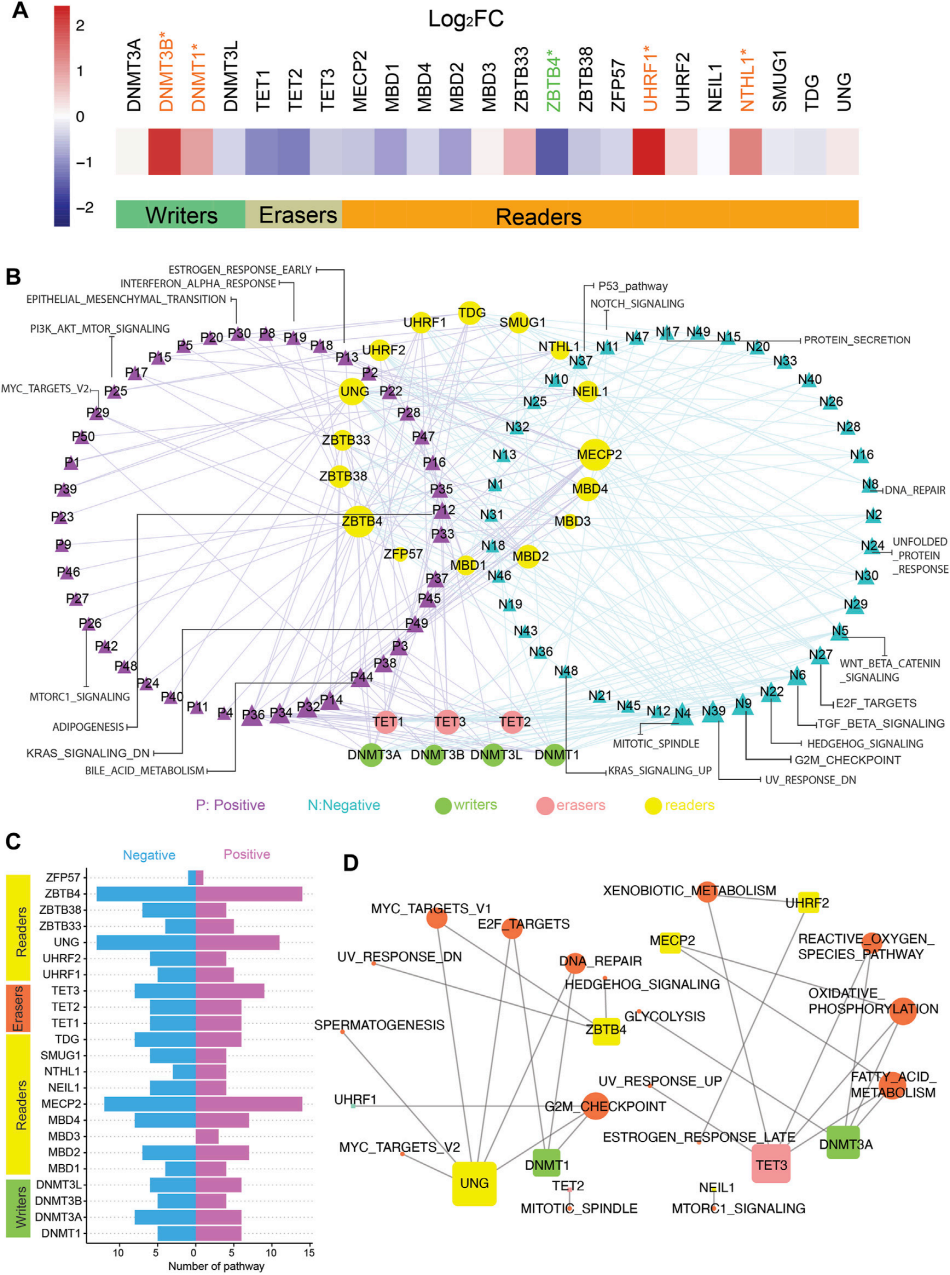
Active and suppressive pathways of cancer are associated with 5mC regulators in colon cancer. **(A)** Gene expression of 5mC regulators in tumor and normal samples. *t*-test was used to evaluate differences between two groups, *p-value < 0.05. **(B)** Network pictogram for hallmark-related pathways and 23 5mC regulators based on GSVA and correlation. Size of nodes is in proportion to number of related links. **(C)** Number of pathways correlated with individual 5mC regulators. (Right: positively correlated pathways; left: negatively correlated pathways.). **(D)** Pathways highly related with 23 5mC regulators (|SCC| > 0.5 and *p*-value < 0.05). Size of nodes is in proportion to number of related links.

We curated a catalog of 23 5mC regulators according to the published research. They could fall into three main categories: writers (DNMT1, DNMT3A, DNMT3B, and DNMT3L), erasers (TET1, TET2, and TET3), and readers (MBD1, MBD2, MBD3, MBD4, MECP2, NEIL1, NTHL1, SMUG1, TDG, UHRF1, UHRF2, UNG, ZBTB33, ZBTB38, ZBTB4, and ZFP57). The gene expression data and matching methylation data were obtained from TCGA (all details were recorded in *Materials and Methods*). Analysis of global gene expression profiles identified the differential expression of these 5mC regulators. Among writers and readers, DNMT1, DNMT3B, UHRF1, and NTHL1 mRNA levels were markedly upregulated in CC patients, whereas the low expression of ZBTB4 was observed in CC patients (Figure 1A). Then, the correlation between the expression of individual regulators and hallmark pathways related to CC was analyzed by GSEA (Figure 1B; Supplementary Table S4). The first two genes, ZBTB4 and MECP2, were involved in most of the pathways, such as the inhibition of apical junction, hedgehog signaling, and NOTCH signaling pathways, and the activation of reactive oxygen species and bile acid metabolism pathways (Figures 1B,C). In addition, it can be seen that different writers, readers, or erasers were bound up with distinct hallmark pathway alterations, which meant disparate roles of 5mC regulators in the identical functional pathway (Figures 1B,C). The expression of ZBTB4, MBD1, and MECP2 positively affected the MYC targets pathway, contrary to DNMT3B and UNG. The positive impact of SMUG1 and UHRF2 on the phosphatidylinositol-3-kinase–AKT–mammalian target of rapamycin signaling pathway was also produced, in contrast to TDG and UNG. DNMT3B and ZBTB33 were positively associated with the P53 pathway, opposite of the influence of MBD2 and MBD1. In particular, DNMT3L, ZBTB38, ZBTB4, MECP2, and all erasers were negatively related to the transforming growth factor-β signaling pathway. DNMT3B, DNMT3A, and DNMT3L were involved in the inhibition of the Wnt/β-catenin signaling pathway.

Following that, we identified the 5mC regulators strongly related to the active or suppressive pathways with |SCC| > 0.5 and adjusted *p*-value < 0.05 (Figure 1D). Strong relativity was found between the DNA repair pathway and DNMT1, UNG, but there was no evidence that DNMT1 has a noticeable effect on DNA repair in this analysis (Figures 1B,D). The expression of DNMT1 and UNG strongly and negatively correlated with the E2F targets pathway (Figures 1B,D).

### Interactions Among 5-Methylcytosine Regulators

**FIGURE 2:**
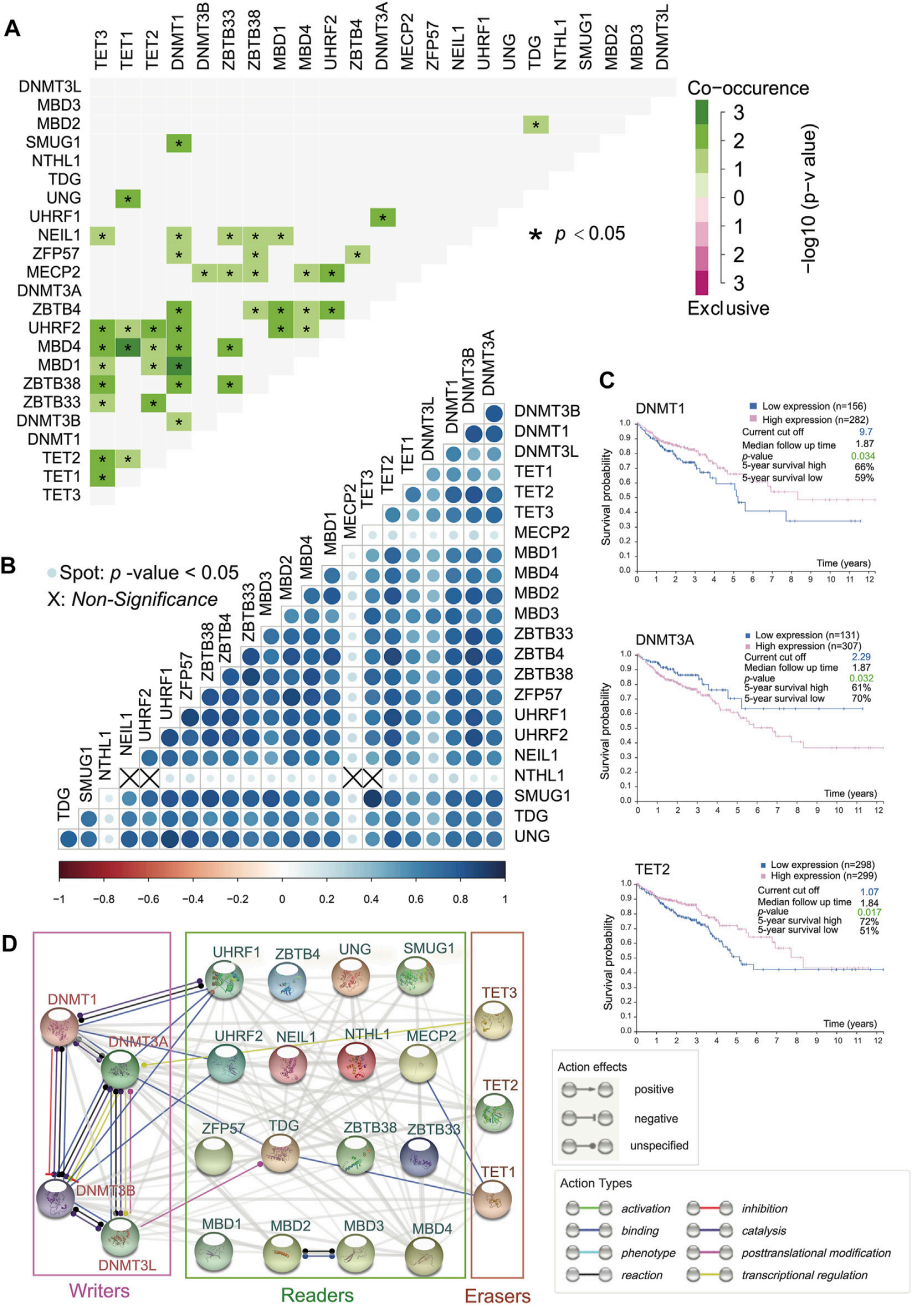
Positive interaction generally among 5mC regulators and survival analysis. **(A)** Somatic mutation interactions among 23 5mC regulators. (*p-value < 0.05). DNMT3L and MBD3 are not related to other 5mC regulators. Other significant relationships are positive. **(B)** SCC regarding expression profile of 23 5mC regulators (Spot: *p*-value < 0.05). Writers are related to erasers and readers. **(C)** Overall survival curves of DNMT3A, DNMT1, and TET2 (*p*-value < 0.05) indicate that all of them are clinically significant. **(D)** Functional protein association network among 5mC regulators.

The interactions among the 23 DNA methylation regulators produced highly correlated behaviors. We analyzed the interaction network among these regulators (Figure 2A) and calculated SCC among them after confirming the violation of normal distribution (Figure 2B). Interestingly, it turned out that DNA methyltransferases exerted synergistic efficacy as writers because of mutual binding action type, whereas proteins involved in DNA demethylation acted independently without binding behavior, but there was a significant positive correlation in writers or erasers due to large SCC (Figures 2A,B). Besides, there were complex network interactions and positive correlations between readers. Additionally, MBD4-TET1, DNMT1-TET2/MBD1, and UHRF1-UHRF2 had strong evidence of co-occurrence in somatic interactions as a result of the high odds ratio of the gene set and a *p*-value < 0.05. Moreover, it was apparent that there was a co-occurrence of genomic mutation between erasers with a *p*-value < 0.05 (Figure 2A). As could be seen from Figure 2B, NTHL1 did not correlate with TET3, UHRF2, NEIL1, and MECP2. MBD family members involved, ZBTB family members involved, and UHRF1, UHRF2, ZFP57, and NEIL1 were positively related to readers and erasers (Figure 2B). It was a special outpouring of attention to a wide range of action types that occurred among writers (Figure 2D). The TDG as a reader, due to its binding action type, seemed to be a bridge between DNMT3A (writer) and TET1 (eraser) (Figure 2D).

### Clinical Significance of 5-Methylcytosine Regulators

The prevalent transcriptional expression alterations of 5mC regulators in CC may provide a vital perspective into clinical utility. To judge the effect of regulators on the overall survival of patients, survival analysis was performed simultaneously, and the optimal cutoff points were obtained from maximally selected rank statistics based on the HPA platform. Besides, target drugs of clinical regulators in clinical trials or markets were collected (Table 1). The result represented three genes with targeted drugs and survival differences, including DNMT3A, DNMT1, and TET2 (Figure 2C). After finding the optimal cutoff value of gene expression by the HPA website, the high expression group of DNMT1 and TET2 predicted better survival than the low expression of them with minor differences in the former and major differences in the latter, yet DNMT3A was the opposite of that result with little distinction. What stands out in Table 1 is that DNMT3A, DNMT1, and TET2 all were affected by decitabine and azacitidine. It is worth mentioning that DNMT1 is a differentially expressed gene with the significant overall survival of patients.

**TABLE 1:** Emphasis on 5mC regulators and corresponding drugs.

Gene	Favorable prognosis	No. drug	Description		Name	
DNMT1	high expression	14	Inhibitor	Decitabine^a^	Azacitidine^a^	CHEMBL2349526
		—	—	Diethylstilbestrol	Hydroxyurea	Ifosfamide
		—	—	Zebularine	Arsenic Trioxide	Floxuridine
		—	—	Adriamycin	MG98	Mitoxantrone
		—	—	Cisplatin	FTI (farnesyltransferase Inhibitors)	—
DNMT3A	low expression	4	Inhibitor	Decitabine^a^	Azacitidine^a^	—
			—	Daunorubicin	Idarubicin	—
TET2	high expression	2	—	Decitabine^a^	Azacitidine^a^	—

a Represents drugs targeted for DNMT1, DNMT3A, and TET2.

### Identification of DNA Methylation-Driven Genes

**FIGURE 3:**
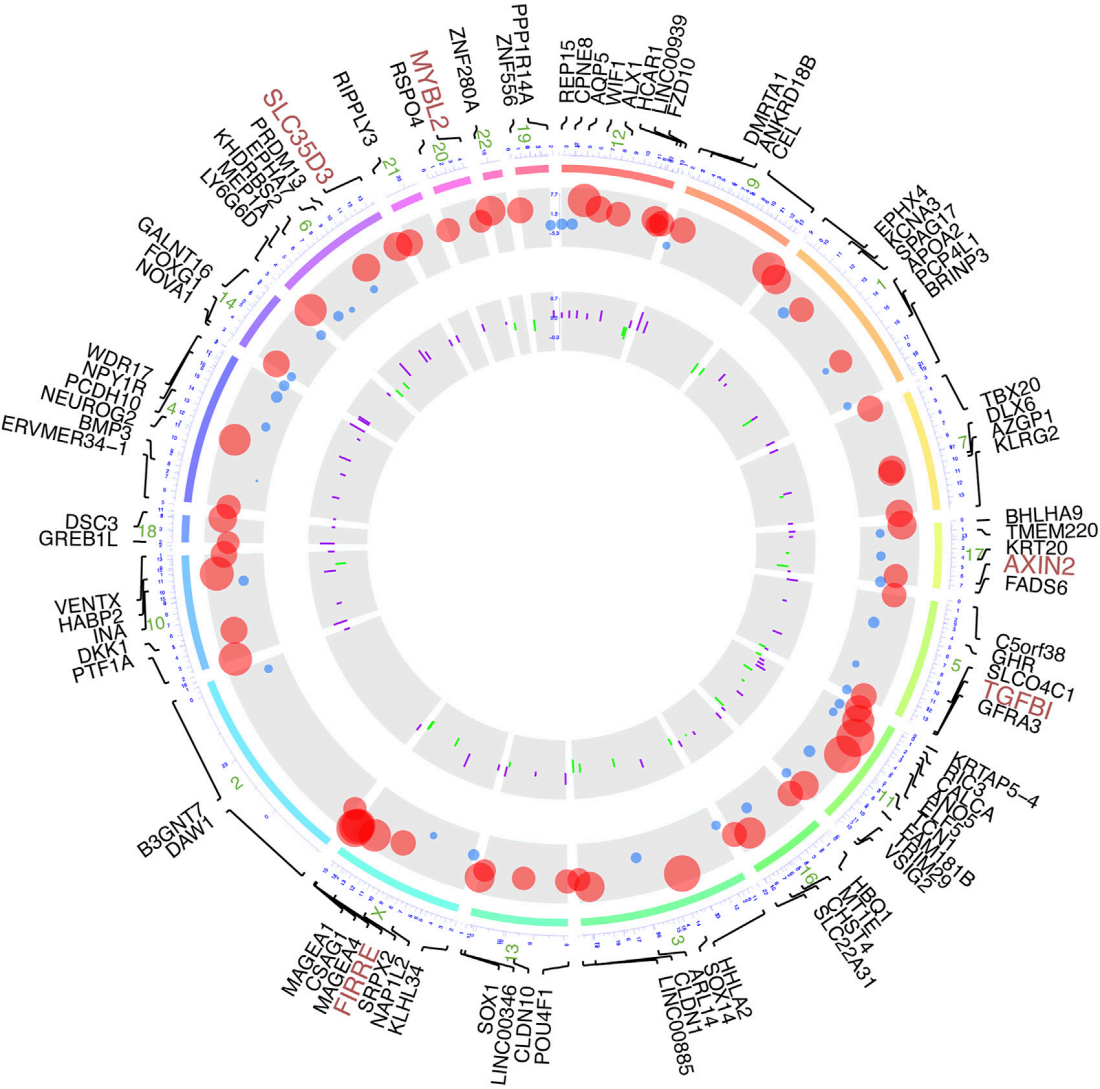
Circos plot of DNA methylation-driven genes based on multi-omics. From outermost circle to inner circle, presentation on map is as follows: chromosome location with lines deriving from specific gene locus, log_2_FC of transcriptome expression by dot plots with size of dots proportional to value and with red showing high expression and blue showing low expression, and log_2_FC of DNA methylation by bar charts provided with purple indicating hypermethylation and green indicating hypomethylation.

Based on the manipulation criteria from the methods pattern, 108 genes (Supplementary Table S1) were defined considering differential methylation level and differential RNA expression level (Figure 3). For the sake of apparent observation of DNA methylation and transcriptome expression differences of those genes screened, we displayed chromosomal location annotation of the corresponding genes selected, DNA methylation degree, and log_2_FC of transcriptome expression collected from TCGA database in a circos plot utilizing BioMart databases (Figure 3). As a whole, the DNA methylation degree tends to be negatively related to the expression level of the corresponding gene. The consequence demonstrated that these genes were distributed in chromosomes other than 5, 8, and Y chromosomes, and chromosome 1 contained the most genes. Those genes with diagnosis significance were highlighted in plum, including AXIN2, FIRRE, MYBL2, SLC35D3, and TGFBI.

### Functional Enrichment Analysis of Methylation-Driven Genes

**FIGURE 4:**
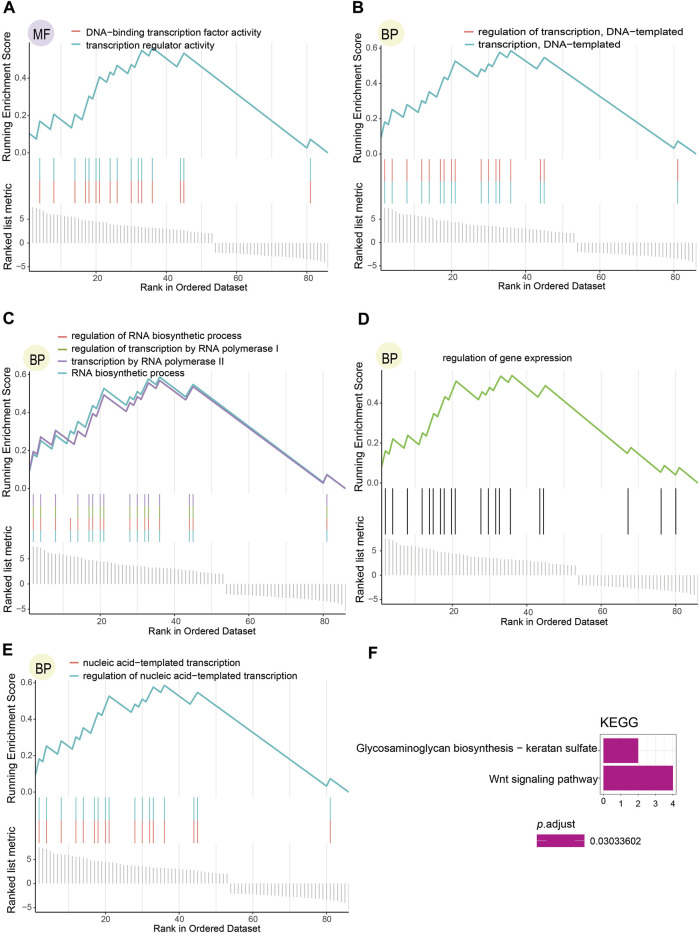
Functional enrichment analysis results regarding DNA methylation-driven genes selected. **(A)** Consequence of molecular functions with *p*.adjust < 0.05. **(B–E)** Outcome of biological processes with *p*.adjust < 0.05. **(F)** Result of KEGG pathways with *p*.adjust < 0.05.

To further inquire about the function of DNA methylation-driven genes screened, we performed the GSEA.GO analysis (Figures 4A–E; Supplementary Table S2) and the KEGG pathway analysis (Figure 4F), which were finally selected per the relevant results with *p*.adjust < 0.05 (*p*-value after adjustment). The results indicated that these genes functioned as physiological regulatory effects were enriched in both BP and MF but not in CC, and only two pathways were enriched in KEGG pathways analysis. Transcription regulator activity and DNA-binding TFs activity were enriched in the MF (Figure 4A). In addition, the outcome enriched in BP mainly brought about the following characteristics: DNA-templated transcription and its regulation (Figure 4B), RNA biosynthetic process and its regulation, transcription by RNA polymerase II and its regulation (Figure 4C), regulation of gene expression (Figure 4D), and nucleic acid-templated transcription and its regulation (Figure 4E). Wnt signaling pathway and glycosaminoglycan biosynthesis with keratan sulfate were enriched as KEGG pathways (Figure 4F.).

### Preliminary Screening for Clinical Evaluation of Methylation-Driven Genes

**FIGURE 5:**
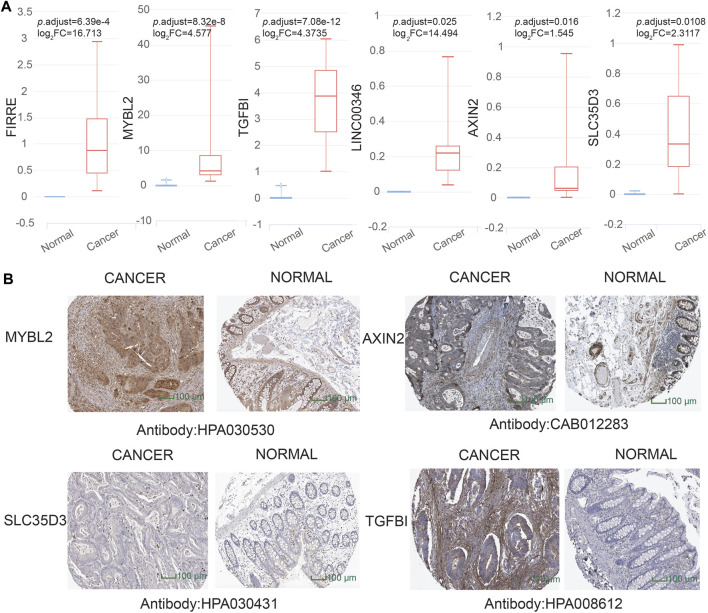
Assessment of preliminary shortlisted candidate gene expression level in extracellular vesicles (EVs) and immunohistochemistry. **(A)** Highly expressed methylation-driven genes (FIRRE, MYBL2, TGFBI, LINC00346, AXIN2, and SLC35D3) in EVs for cancer samples. **(B)** Higher protein expression of immunohistochemistry (MYBL2, AXIN2, TGFBI, and SLC35D3) in cancer tissue than normal tissue from the Human Protein Atlas (HPA) project database (https://www.proteinatlas.org/).

**FIGURE 6:**
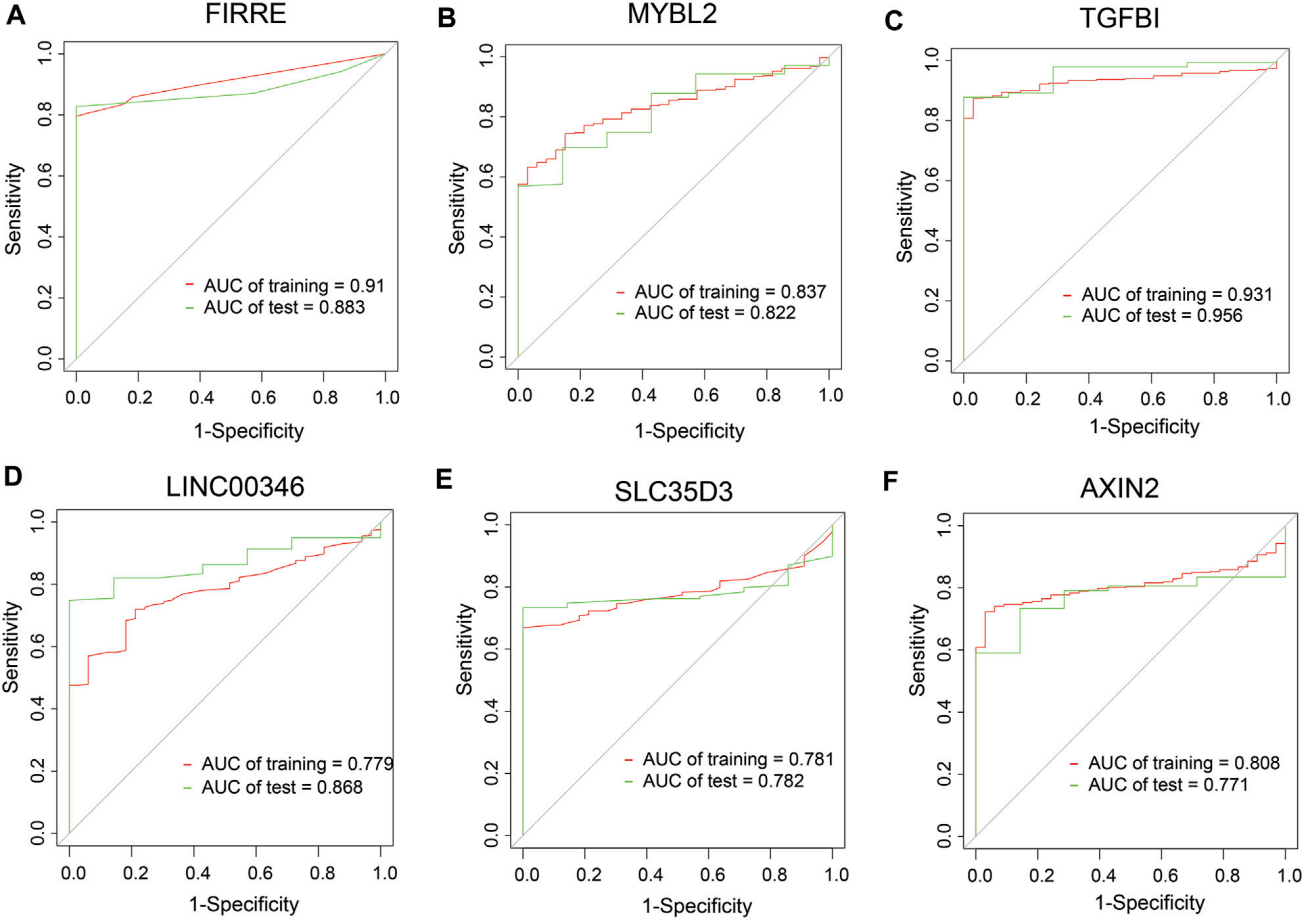
Clinical performance assessment of preliminary shortlisted genes. **(A–F)** ROC curves of single genes, including FIRRE, MYBL2, TGFBI, LINC00346, SLC35D3, and AXIN2. AUC values are greater than 0.7.

To dig out the application in clinical diagnosis, we initially assessed the level of gene expression in EVs (Figure 5A) *via* the BBCancer dataset depending on *p*.adjust < 0.05 and |log_2_FC| > 1.5 (i.e., RNA expression degree) and immunohistochemistry *via* the HPA dataset (Figure 5B) (i.e., protein expression degree), and then related genes was identified with the AUC of more than 0.7 (Figure 6). Concerning the proviso discussed, two long noncoding RNAs (FIRRE and LINC00346) and four protein-coding RNAs (AXIN2, MYBL2, TGFBI, and SLC35D3) were worthy of selection. FIRRE and LINC00346 had significant expression differences in EVs with |log_2_FC| > 10 and *p*.adjust < 0.05, with other genes' expression having differences in EVs (Figure 5A). Immunohistochemical staining was performed for AXIN2, MYBL2, TGFBI, and SLC35D3, which represented that the part of cancer was deeper than that of normal (Figure 5B). All in all, the expression profile result of RNA and protein showed that all candidate genes were positively expressed in patients with CC. To identify the potential gene signature, AUC analysis was performed, dependent on a single gene. TCGA samples were divided into the training set and the test set (training set–test set = 7:3). The assessment result of a single gene indicated that for all genes, the test was close to the training. The values of the test set for FIRRE, MYBL2, TGFBI, LINC00346, SLC35D3, and AXIN2 were 0.833, 0.822, 0.956, 0.868, 0.782, and 0.771, respectively (Figures 6A–F). Furthermore, the values of the training set for FIRRE, MYBL2, TGFBI, LINC00346, SLC35D3, and AXIN2 were 0.910, 0.837, 0.931, 0.779, 0.781, and 0.808, respectively (Figures 6A–F).

### Somatic Variants of 5-Methylcytosine Regulators and Preliminary Shortlisted Methylation-Driven Genes

Somatic mutation can be divided into three major types, point mutation, chromosomal mutation, and genomic mutation. Herein, we analyzed somatic genomic mutation and CNAs. We identified 30.58% of patients who experienced somatic genomic mutations of 5mC regulators, comprising frameshift insertions or deletions, in-frame deletions, multi-hit, etc. (Supplementary Figure S1A). Despite a large number of patients in genomic mutation, only erasers (TET1, TET2, and TET3) were over 5% (Supplementary Figure S1A). As far as CNAs, multiple characters were observed among regulators. In the readers, it indicated that the most frequent was DNMT3B, which occupied 73% amplification or gain among all regulators. DNMT3A (21%), DNMT1 (26%), and DNMT3L (35%) all had loss and gain in CNAs, notably with the highest frequency of loss in DNMT3L. For erasers, TET1 (25%) and TET2 (38%) mainly manifested loss, yet the gain of TET3 (20%) predominated. With the CNV rate of over 50% MBD1, MBD2, and ZBTB4, they focused on more loss and less gain. However, the gain of other readers was more than loss among patients (Supplementary Figure S1C).

Concerning somatic genome alteration of prospective diagnosis genes, AXIN2 altered in 7% of 399 samples, whereas the alteration rate of MYBL2, TGFBI, SLC35D3, FIRRE, and LINC00346 accounted for less than 5%. In CNAs of them, MYBL2 was the top first gene that occupied 72% for the alteration pattern of gain and amplification, and LINC00346 was the top second gene that occupied 61% for that of gain and amplification in the majority. TGFBI, SLC35D3, and AXIN2 were lower than 50%; moreover, there were no data of FIRRE (Supplementary Figure S1).

### Function Analysis of Preliminary Shortlisted Genes

To further inquire about the function of shortlisted genes discussed earlier, GO analysis and KEGG analysis were performed, and all the significant results were displayed (Supplementary Figures S2A–D). The results of KEGG analysis showed that multiple cancer induction, particularly CRC and signaling pathways including Wnt and Hippo, was enriched (Supplementary Figure S2A). In MF, different binding activities were witnessed, such as 1-SMAD binding and extracellular matrix binding (Supplementary Figure S2B). For CC, the consequent revealed the β-catenin destruction complex and basement membrane (Supplementary Figure S2C). As for BP, chondrocyte differentiation, cartilage development, and connective tissue development were enriched (Supplementary Figure S2D).

### Interactions Between Preliminary Shortlisted Genes and Transcription Factors

**FIGURE 7:**
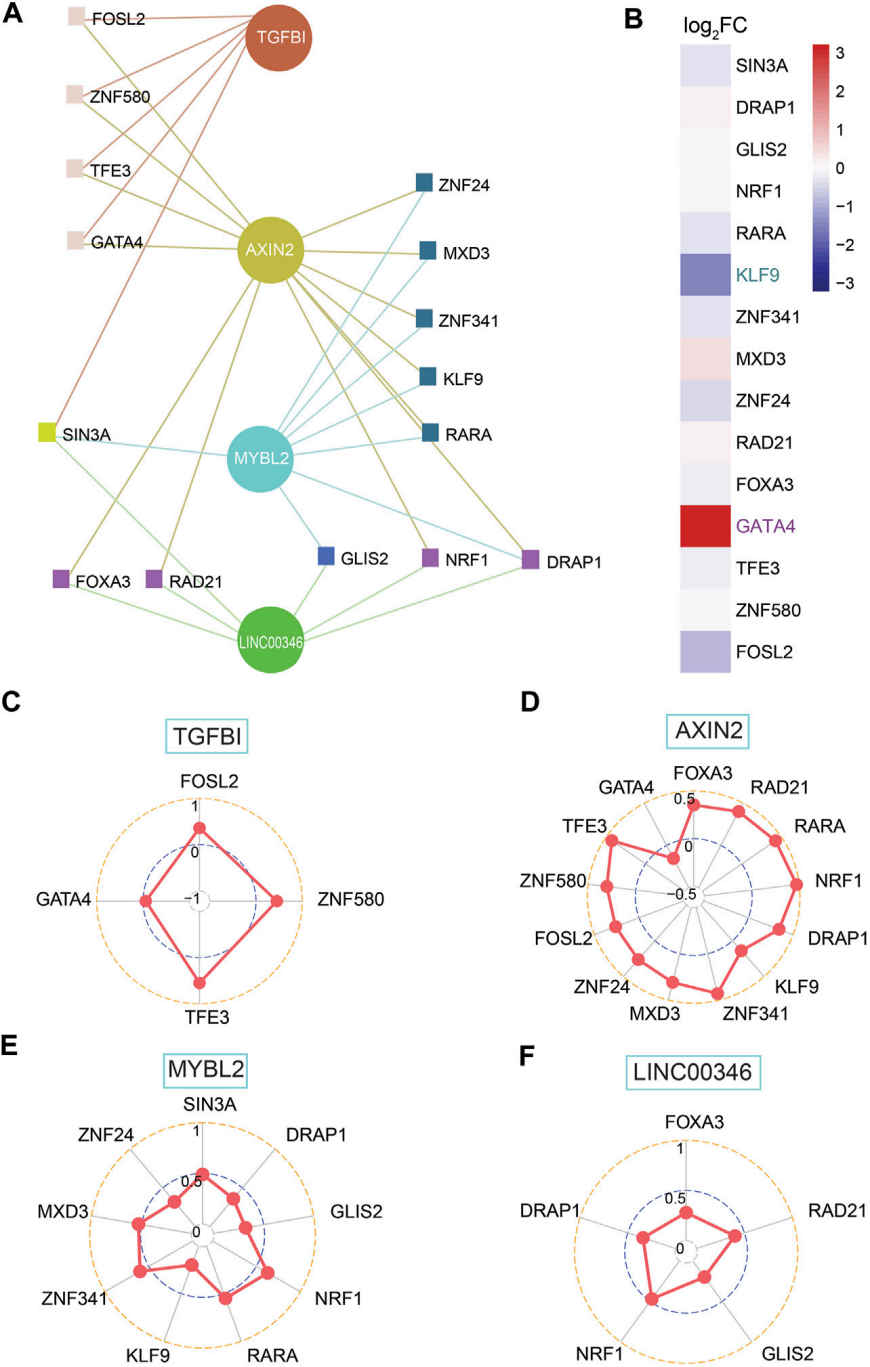
Investigation of shortlisted genes and transcription factors (TFs) together with correlation among them. **(A)** Network of shortlisted genes and TFs. **(B)** Heatmap of log_2_FC for RNA expression of 15 TFs (high expression: GATA4; low expression: KLF9, both of them is |log_2_FC| > 1.5). **(C–F)** SCC between TFs and genes, including TGFBI, AXIN2, MYBL2, and LINC00346, based on network among them.

TFs, as a direct interpretation of the genome, are one of the most significant components in performing DNA decoding sequences. Thus, we moved on to the further step to work out dependent molecular regulating the expression of shortlisted genes involving AXIN2, FIRRE, LINC00346, MYBL2, SLC35D3, and TGFBI. Further analysis shows that AXIN2, LINC00346, MYBL2, and TGFBI had a network with TFs (Figure 7A). Next, we screened out TFs, which implied they were able to be regarded as transcription activators such as MXD3 and SIN3A or transcriptional repressors such as GATA4, which embodied KLF9 and zinc finger protein consisting of ZNF24, ZNF580, GLIS2, ZNF341, etc. (Figure 7A). Only high expression of GATA4 (log_2_FC = 4.43), together with low expression of KLF9 (log_2_FC = −2.11), was geared to differential expression genes with |log_2_FC| > 1.5 (Figure 7B; Supplementary Table S3). All genes were associated with their relevant TFs for a *p*-value < 0.05 (Figures 7C–F). There was a positive correlation between TGFBI and three TFs covering FOSL2, TFE3, and ZNF580, whereas a negative correlation to GATA4 (Figure 7C). AXIN2 was positively related to TFs except for GATA4 (Figure 7D). A strongly positive correlation was found between MYBL2 and TFs involving NRF1, RARA, MXD3, and ZNF341 (Figure 7E). LINC00346 was positively related to FOXA3, RAD21, GLIS2, NRF1, and DRAP1 (Figure 7F).

### Evaluation and Verification of the Diagnostic Signature of Methylation-Driven Genes

**FIGURE 8:**
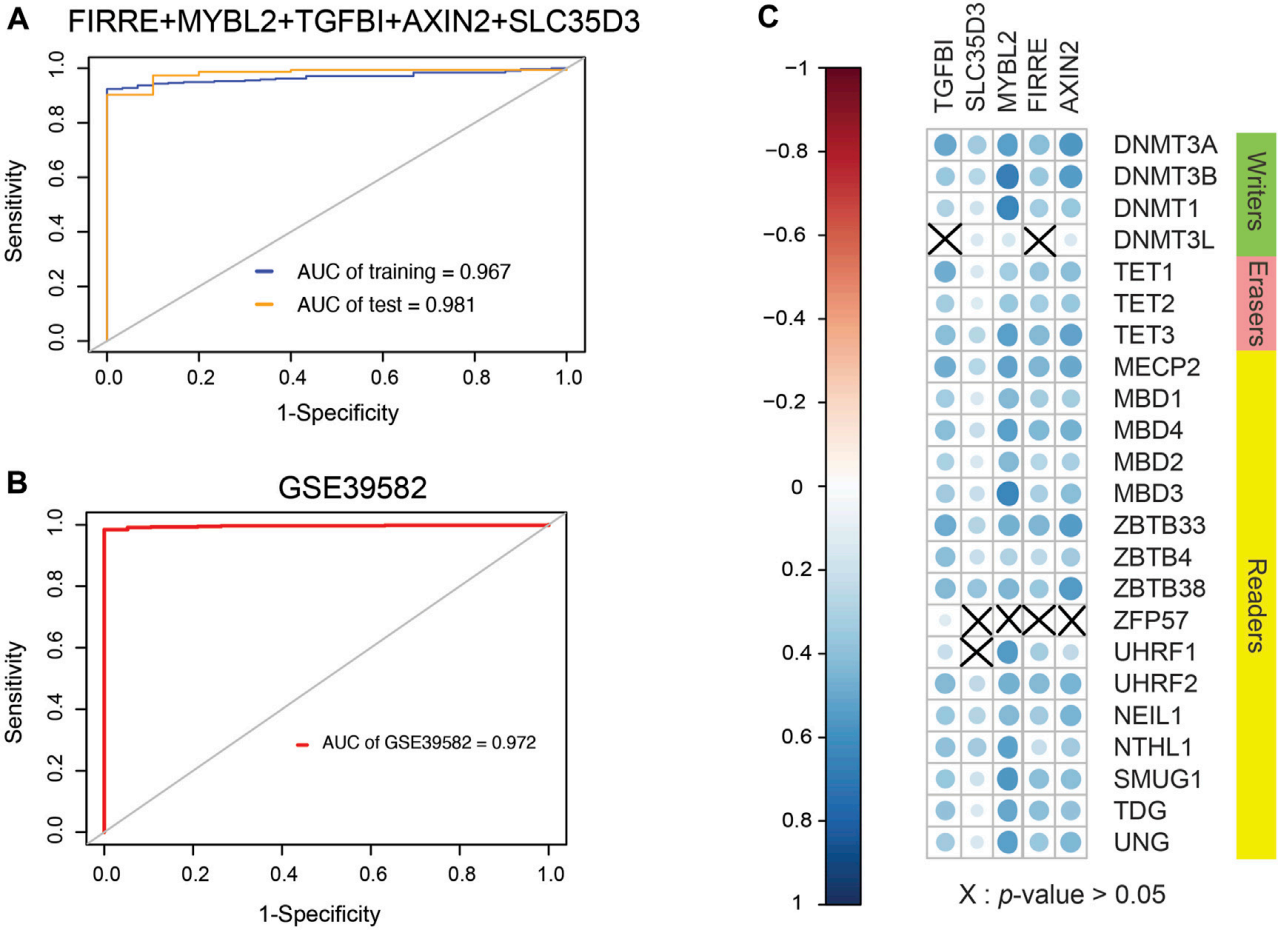
Clinical performance assessment of signature and Spearman correlation coefficient between them and 5mC regulators. **(A)** ROC curves of five-gene signature involving FIRRE, MYBL2, TGFBI, AXIN2, and SLC35D3 (AUC of training set = 0.967, AUC of test set = 0.987). **(B)** ROC curves of diagnostic signature in GSE39582, which includes 19 normal cohorts and 566 cancer cohorts (AUC of GSE39582 = 0.972). **(C)** SCC between them and 5mC regulators. Size of nodes is inversely proportional to *p-*value. Erasers are related to genes from signature, and a part of writers and readers are related to these genes.

Because EVs contain signal molecules such as protein, mRNA, microRNA, etc., they can often be used to reflect the physiological and pathological functional statuses of secreting cells, thereby providing potential biomolecular markers, so the purification of exosomes from body fluids has a good effect. Clinical application prospects. The tumor will continuously release exosomes into the surrounding environment during the growth process, so searching for differentially expressed genes of EVs has potential clinical significance. After comparing gene expression differences in EVs based on the BBCancer database by the “limma” package and the property of universal probes, mixed genes comprising FIRRE, MYBL2, TGFBI, AXIN2, and SLC35D3 were selected. By fitting generalized linear model using iteratively reweighted least-squares (Supplementary Table S5) based on preliminary shortlisted genes, the conclusive diagnostic model formula (AIC = 134.88) is 0.9539 × FIRRE + 0.4983 × MYBL2 + 1.2050 × TGFBI − 0.3430 × AXIN2 + 0.1281 × SLC35D3. Surprisingly, the AUC values of the integration were 0.987 in the test set and 0.967 in the training set, which is larger than single genes in TCGA database (Figure 8A). The optimal cutoff value is 16.2736569. To validate the model, GSE39582 was collected with 19 normal cohorts and 566 cancer cohorts. The AUC value of this model in the GSE39582 is 0.972, which supports the clinical significance of our model (Figure 8B).

### Interactions Between Methylation-Driven Genes and 5-Methylcytosine Regulators

DNA methylation is a form of DNA chemical modification that can change the activity of a DNA piece without transforming the sequence and is regulated by DNA methylation regulators. Given inquiry into the relevance between 5mC regulators and genes selected, SCC was formulated (Figure 8C). As a consequence, a positive correlation was found between AXIN2 and regulators except for ZFP57. FIRRE was positively associated with regulators other than DNMT3L and ZFP57. There was a positive correlation between MYBL2 and regulators except for ZFP57. SLC35D3 showed a positive correlation with regulators excluding ZFP57 and UHRF1. TGFBI represented a positive interaction of regulators apart from DNMT3L and UHRF1. Generally, 5mC regulators interfere with the expression of DNA methylation-driven genes according to SCC.

## Discussion

Epigenetic processes are important mechanisms in the development of CC. The clinical value of epigenetic markers has been demonstrated. In this study, differential expression analysis of 5mC regulators between cancer and normal tissues was performed. The relationship between the regulators and hallmark-related pathways, which manifested that MYC targets pathway, phosphatidylinositol-3-kinase–AKT–mammalian target of rapamycin signaling pathway, and P53 pathway were subject to the influence of regulators' expression, was identified. Then, we investigated the interaction among 5mC regulators in CC in somatic interaction and expression interaction. Survival analysis indicated DNMT3A, DNMT1, and TET2 associated with the prognosis of the patients with CC. Furthermore, DNA methylation-driven genes in CC were all identified. In general, the degree of DNA methylation appears to be negatively correlated with the expression level of the corresponding DNA methylation-driven gene after observation of expression level in EVs and histology. We developed a five gene signature (FIRRE, MYBL2, TGFBI, AXIN2, and SLC35D3) for prospective CC diagnosing. Meanwhile, these genes are positively related to 5mC regulators and interact with their relevant TFs. Validation of this model in EVs in the future will have important implications for CC diagnosis screening.

Abnormal DNA methylation usually occurs during carcinogenesis and has clinical value in human cancer. With the development of methylation sequencing technology, epigenetic changes are easily identified with the sequencing depth and accuracy, including methylation changes and methylation driver events detection. In 2020, a risk score prediction model based on the composition of differentially expressed genes driven by DNA methylation was identified and verified, which can accurately predict the prognosis of gastric cancer in clinical practice (Bai et al., 2020). A recent study constructed a predictive signature of 10-gene methylation in endometrial cancer (Li et al., 2021b). A group of methylation driver protein-coding genes and noncoding RNAs have been identified and experimentally confirmed in lung cancer that could be potential targets for epigenetic therapy by integrating methylation and mRNA expression profile data (Cai et al., 2021). Recently, the domestic team systematically demonstrated a multigene methylation detection method (ColonAiQ) that can realize early screening and recurrence prediction of CC, which is based on six markers for multisite blood detection (Sun et al., 2021). Polygene methylation is expected to become a new “tumor marker” to solve the problems of early screening and recurrence monitoring of cancer.

The long noncoding RNA FIRRE locus is associated with two interrelated features of the inactive X chromosome, namely being located near the nucleolus and maintaining H3K27me3 methylation (Yang et al., 2015). Also, there is a differential methylated region associated with multiple sclerosis in the FIRRE (Souren et al., 2019). The mutation and abnormality of AXIN2 (Axis inhibition protein 2) can lead to the occurrence of ampullary carcinoma (Hayata et al., 2021), endometrial cancer (Syed et al., 2020), and liver cancer (Abitbol et al., 2018) by adjusting the stability of beta-catenin. Ren et al. (2015) discovered that the downregulation of MYBL2 was able to suppress cell proliferation, regulate the cell cycle, and induce apoptosis, which may be involved in mediating aggressive CRC biology. TGFB1, the downstream protein of the transforming growth factor-β signaling, contributes to their metastatic potential and stromal cell independence directly in CRC cells (Chiavarina et al., 2021). Researchers identified SLC35D3 as a new biomarker with prognostic value (Olsson et al., 2020).

In conclusion, we investigated the functions of 5mC regulators in CC systematically. They are related to multiple active and inhibitory pathways associated with CC. Also, we identified the methylation-driven genes from RNA-seq data and matched methylation seq data. Based on such methylation-driven genes, a risk model for diagnosis in CC was developed. Our research would provide new insights into the epigenetic-driven events in CC.

## Data Availability

The datasets presented in this study can be found in online repositories. The names of the repository/repositories and accession number(s) can be found in the article/Supplementary Material.

## Supplementary Material

Additional file 1: Supplementary Figure S1 Somatic genome alteration and CNAs of m5C regulators and shortlisted genes in CC.

Additional file 2: Supplementary Figure S2 KEGG and GO analysis of shortlisted genes.

## Abbreviations

AUC, area under the receiver operating characteristic curve; CC, colon cancer; CRC, colorectal cancer; CNAs, copy number alterations; DEGs, differential expression genes; EVs, extracellular vesicles; SCC, Spearman Correlation Coefficient; TFs, transcription factors; TCGA, The Cancer Genome Atlas; 5mC, 5-methylcytosine.
