# Supplementary figures and images for: Analysis of 5-Methylcytosine Regulators and DNA Methylation-Driven Genes in Colon Cancer
Source: Front Cell Dev Biol. 2022 Jan 31;9:657092. doi: 10.3389/fcell.2021.657092 (PMC8842075; doi:10.3389/fcell.2021.657092)

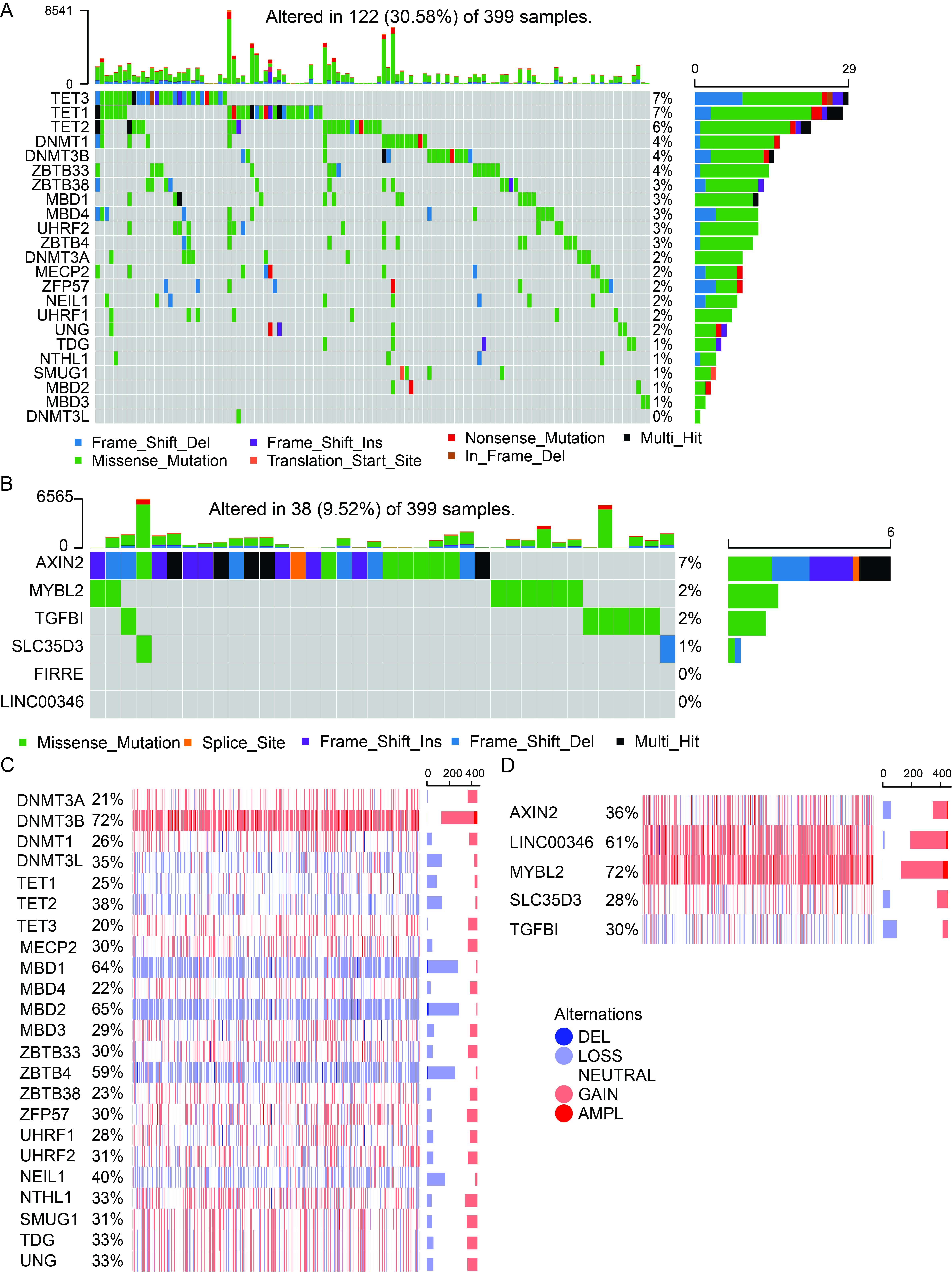

Supplement: Supplementary file 1 [file Image1.JPEG]

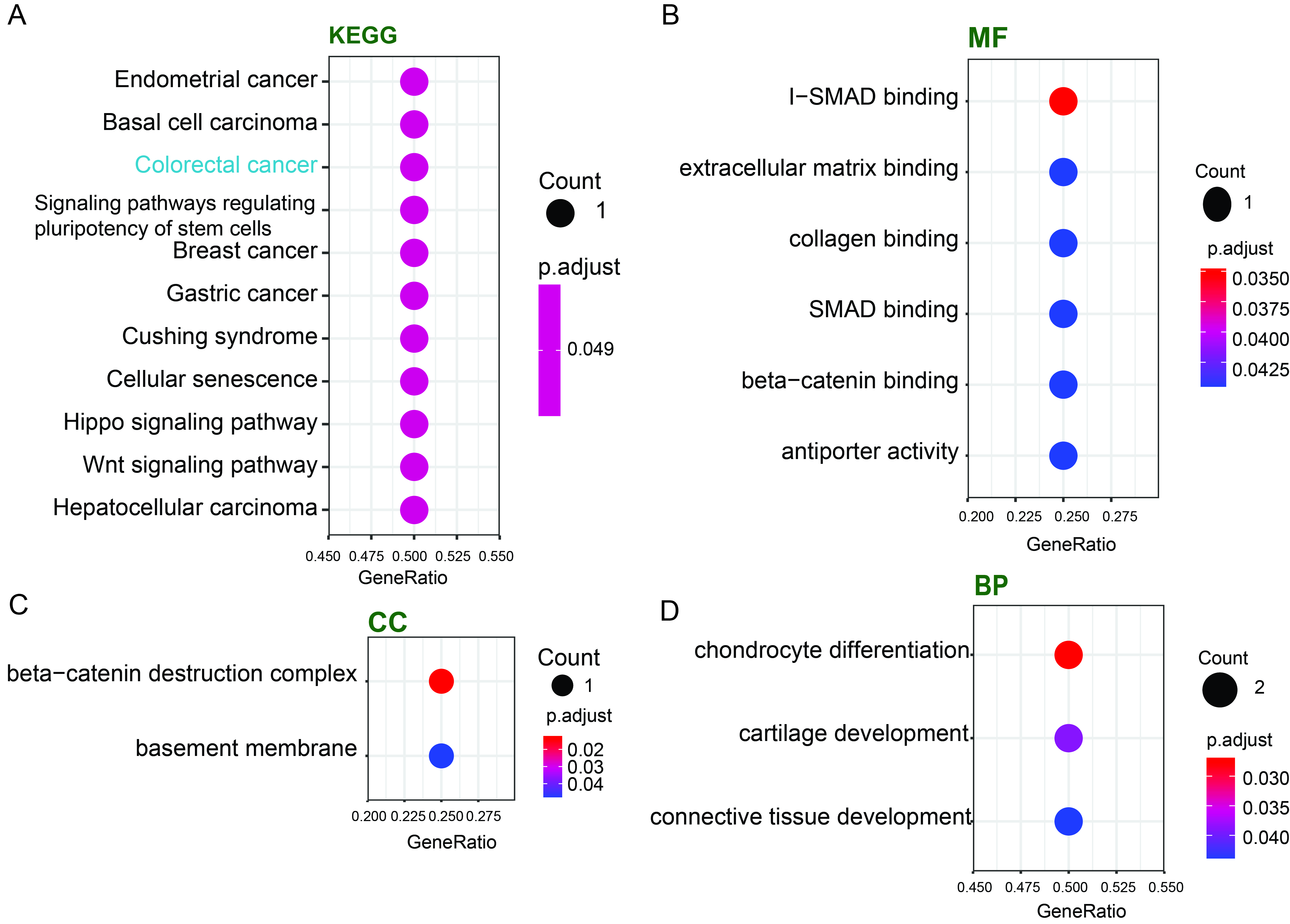

Supplement: Supplementary file 2 [file Image2.JPEG]
